# Association between body roundness index and perceived stress in Chinese adults: A cross-sectional study

**DOI:** 10.1097/MD.0000000000049882

**Published:** 2026-07-24

**Authors:** Naihe Zhang

**Affiliations:** a School of Marxism, Liaoning Engineering Vocational College, Tieling, Liaoning, China.

**Keywords:** adults, body roundness index, cross-sectional study, perceived stress

## Abstract

The objective of this research was to explore the association between perceived stress (PS) levels and the body roundness index (BRI) among adults and older adults in China. Using cross-sectional data extracted from the 2015 China Health and Nutrition Survey, this study analyzed information from 8315 Chinese adults. The 14-item Perceived Stress Scale was used to measure PS, and participants were categorized into high- and low-stress groups based on the median score. Logistic regression analyses were used to assess the relationship. Restricted cubic spline analyses were further performed to explore the potential dose–response relationship and assess the linearity of the association between BRI and perceived stress. Receiver operating characteristic curves were constructed to examine the effectiveness of BRI as an indicator at the individual level. Participants in the highest quartile of BRI had higher odds of elevated PS compared with those in the lowest quartile (fully adjusted odds ratio = 1.35; 95% confidence interval: 1.12–1.62; *P* = .001). Subgroup analyses indicated that the association between BRI and PS was largely consistent across demographic and lifestyle characteristics. No significant interactions were observed, except for smoking status (*P* for interaction = .033), with stronger associations identified among smokers. Sensitivity analyses using a generalized linear model yielded consistent results. Restricted cubic spline modeling identified a linear relationship (*P* = .677), with an apparent rise in PS risk when BRI values exceeded 3.82. Receiver operating characteristic analysis showed limited predictive ability, with area under the curve values ranging from 0.52 to 0.55. This study indicated that elevated BRI was significantly associated with PS among Chinese adults. However, the limited discriminative performance of BRI indicates that this association should not be interpreted as evidence of effective screening capability. Further longitudinal studies are warranted to validate these findings and elucidate the mechanisms underlying this association.

## 1. Introduction

Perceived stress (PS) represents a crucial psychological construct and serves as an essential indicator of mental well-being. It reflects the extent to which individuals perceive life situations as unpredictable, uncontrollable, and overwhelming, and is therefore widely used in epidemiological and psychological research to evaluate stress-related mental health status. Global evidence suggests that approximately one-third of adults experience stress, with prevalence estimates around 35% based on large-scale international surveys and potentially exceeding 40% during major public health crises.^[1]^ Consistent with these findings, large-scale population-based studies in China have reported a substantial burden of PS, with approximately 30% to 50% of adults experiencing elevated stress levels.^[2]^ Given its high prevalence, PS has become a major public health concern. Importantly, PS has been consistently associated with a range of adverse health outcomes, including cardiovascular disease,^[3]^ the development of type 2 diabetes,^[4]^ clinical depression,^[5]^ and cognitive impairment.^[6]^ These findings underscore the importance of identifying determinants of PS, particularly modifiable factors, to inform effective prevention strategies and reduce its associated health burden.

In recent years, increasing attention has been directed toward physiological and metabolic factors that may influence psychological health.^[7]^ The body roundness index (BRI), a novel anthropometric measure of visceral adiposity derived from waist circumference and height,^[8]^ has been increasingly used as a marker of cardiometabolic risk^[9–12]^ and may provide a more accurate reflection of fat distribution than traditional indicators such as body mass index (BMI).^[8]^ Emerging evidence further suggests that BRI may also be linked to psychological outcomes, particularly depression.^[13]^ However, its association with PS remains unclear. Importantly, PS reflects an early-stage psychological response within the stress-disease continuum, capturing subjective stress experiences prior to the onset of clinically diagnosed mental disorders.^[14–16]^ Therefore, examining the association between BRI and PS may provide insight into early interactions between metabolic health and psychological well-being.

Accordingly, this study aimed to investigate the association between BRI and PS in adults, and we hypothesized that higher BRI levels would be associated with increased PS.

## 2. Materials and methods

### 2.1. Study population

Prior publications^[17]^ provide comprehensive explanations of the China Health and Nutrition Survey procedures and methods. North Carolina State University and the Chinese Center for Disease Control and Prevention’s National Institute of Nutrition and Food Safety have been working together on the survey for quite some time.

This study’s data came from the 2015 China Health and Nutrition Survey, which was the first survey wave to include measures of PS. Following the application of completeness and validity criteria to the data, the initial cohort size was reduced from 20,226 to 12,312. The following variables were also excluded: gender (n = 1), age below 18 years (n = 2), implausible mid-upper arm circumference values (n = 208), abnormal BMI records (n = 43), PS (n = 2060), educational background (n = 1617), alcohol consumption (n = 29), smoking status (n = 22), BMI (n = 15), and participants with implausible smoking status. As a result, 8315 participants aged 18 to 94 years were included in the final analysis.

### 2.2. Assessment of PS

To assess the reliability and cultural applicability of the instrument, we used the 14-item Perceived Stress Scale, which has been culturally modified and linguistically validated for use with Chinese communities. This helped us measure PS accurately.^[18]^ From 1 (never) to 5 (very often), respondents used a 5-point Likert scale to score each item. There are 2 parts to the scale: one that measures stress-related distress (items 1, 2, 3, 8, 11, 12, and 14) and another that measures perceived coping capacities (items 4, 5, 6, 7, 9, 10, and 13).^[18]^ Scores for positively worded items ranged from 14 to 70 once they were reverse-scored; greater totals indicated more stress. The scale’s internal consistency in this research was good, with Cronbach’s α = 0.824. For logistic regression analysis, participants’ PS levels were dichotomized at the median, classifying them as either high or low stress, in line with prior research on Chinese populations.^[14,19]^

### 2.3. BRI

The BRI was used to assess body shape ellipticity and abdominal fat distribution. BRI is a dimensionless indicator that usually ranges from 1 to 12. It provides a geometric estimate of body shape. Higher BRI values indicate a more circular body shape, which is associated with greater visceral adiposity and cardiometabolic risk.^[8]^ Anthropometric data collection included waist circumference measurements at the umbilicus and height measurements, recorded to the nearest 0.1 cm using standardized procedures. BRI was computed based on an established equation.^[8,11]^


$$BRI=364.2-365.5\;\times \;\sqrt{1-{\left(WC\;/\;\left(2\pi H\right)\right)}^{2}}$$

WC denotes waist circumference (cm), and *H* denotes height (cm).

Participants were further divided into quartiles based on the distribution of BRI values, defined as Q1 (lowest BRI), Q2, Q3, and Q4 (highest BRI).

### 2.4. Covariates

Participants’ weights were recorded to the nearest 0.1 kg utilizing a precisely calibrated electronic scale, with subjects dressed in lightweight clothing. Height measurements were taken without footwear using a portable stadiometer, accurate to 0.1 cm. BMI was derived by dividing weight in kilograms by the square of height in meters (m^2^),^[20]^ and participants were categorized based on World Health Organization guidelines into BMI groups of <30 kg/m^2^ or ≥30 kg/m^2^.^[21]^ Trained staff members collected demographic and health behavior information using standardized surveys. Variables included age classification,^[22]^ sex, level of education, type of residence, geographical area, smoking status, and alcohol use history.

### 2.5. Statistical analysis

Descriptive analyses of participant characteristics were conducted across quartiles of BRI. Continuous data were expressed as mean values accompanied by their 95% confidence intervals (CIs) and compared using one-way analysis of variance, while categorical variables were summarized as frequencies and percentages and compared using chi-square (χ^2^) tests.

To examine the association between BRI and PS, 2 types of regression models were applied according to the nature of the PS outcome. When PS was treated as a continuous variable (14-item Perceived Stress Scale score), generalized linear models were used to estimate β coefficients and corresponding 95% CIs, allowing assessment of linear relationships between BRI and PS levels. When PS was dichotomized into high- and low-stress groups based on the median score, binary logistic regression models were employed to estimate odds ratios and 95% CIs using maximum likelihood estimation. This dual-model approach was adopted to assess the robustness of the findings across different outcome specifications.

Subgroup analyses were performed to explore potential effect modification across demographic and lifestyle factors, including sex, age, BMI, smoking status, drinking status, residential area, geographic region, and educational level. The discriminative ability of BRI to identify elevated PS was evaluated using receiver operating characteristic curves, with the dichotomized PS variable as the outcome. The area under the curve (AUC) was calculated, with higher values indicating better discrimination. Restricted cubic spline (RCS) regression with knots at the 10th, 50th, and 90th percentiles of BRI was used to explore potential nonlinear associations between BRI and PS. All statistical analyses were conducted using Stata version 16.0 (StataCorp LLC, College Station), and a two-sided *P*-value <.05 was considered statistically significant.

## 3. Results

Table 1 presents a summary of the baseline characteristics of participants across the quartiles of the BRI. Overall, a distinct gradient pattern was discerned for several crucial variables. Individuals with elevated BRI levels tended to be older and have higher BMI values, accompanied by a gradually increasing burden of cardiometabolic comorbidities, including hypertension and diabetes. The distribution of sex varied across the quartiles, with a relatively higher proportion of males observed in the middle groups. Lifestyle behaviors such as smoking and alcohol consumption also differed among the quartiles, although these patterns did not adhere to a strictly monotonic trend. Regarding residential and socioeconomic characteristics, higher BRI levels were generally associated with a greater representation of urban residents and individuals with lower educational attainment, particularly those with primary education. The geographic distribution also showed variation, with higher BRI levels corresponding to a higher proportion of participants from the eastern regions and a lower proportion from the western regions.

**Table 1:** Participant characteristics according to quartiles of BRI.

Variable	Quartile 1	Quartile 2	Quartile 3	Quartile 4	χ^2^/*F*	*P* for trend*
Age range, yr	18–86	18–94	18–94	18–92	–	–
Mean value of BRI	2.4 ± 0.5	3.4 ± 0.2	4.3 ± 0.3	5.6 ± 0.8	–	–
Age, yr	45.3 (44.6, 45.9)	50.1 (49.5, 50.6)	52.8 (52.3, 53.4)	55.78 (55.2, 56.3)	221.7	<.001
Sex (male), %	46.0	53.0	52.0	45.0	41.4	<.001
Smoker, %	26.4	28.7	28.1	23.8	15.4	.002
Drinker, %	26.5	30.1	31.8	27.2	18.4	<.001
Residential regions, %					30.4	<.001
Urban	24.7	26.9	27.5	27.9		
Suburban	13.7	14.4	15.6	15.5		
County	17.4	19.6	20.2	19.0		
Rural	44.2	39.1	36.8	37.6		
BMI, kg/m^2^	20.9 (20.8, 21.0)	23.4 (23.3, 23.4)	25.21 (25.1, 25.3)	27.66 (27.5, 27.8)	2723.8	<.001
Educational level, %					174.5	<.001
Primary school	14.7	18.0	18.8	26.2		
Junior high school	37.2	38.4	39.2	38.0		
Senior high school	16.6	17.8	17.9	15.5		
Vocational school	9.5	10.0	9.3	9.2		
College	20.8	15.1	14.4	11.0		
Master’s degree or above	1.2	0.9	0.4	0.1		
Geographic region, %					95.5	<.001
Eastern	33.7	34.5	37.9	36.2		
Central	24.4	23.9	23.0	24.8		
Western	29.5	23.6	21.4	19.3		
Northeastern	12.4	18.0	17.7	19.7		
Comorbidities, %						
Hypertension	6.4	12.0	18.2	27.3	372.1	<.001
Diabetes	1.8	3.9	5.5	8.0	91.6	<.001

ANOVA = analysis of variance, BMI = body mass index, BRI = body roundness index.

* Comparisons between groups were performed using one-way ANOVA for continuous variables and chi-squared tests for categorical variables.

The positive association between BRI and PS is presented in Table 2 and remained robust after full adjustment. A clear dose–response pattern was observed across BRI quartiles. In the fully adjusted model, individuals in the highest quartile had a 35% higher risk of PS compared with those in the lowest quartile (odds ratio = 1.35, 95% CI: 1.12–1.62; *P* for trend = .001), supporting an independent and graded association between BRI and PS.

**Table 2:** Logistic regression analysis of the association between BRI and risk of PS.

N = 8315	Total participants	Number of PS	Model 1*	Model 2†	Model 3‡
Quartile 1	2079	1096	1.00 (Reference)§	1.00 (Reference)§	1.00 (Reference)§
Quartile 2	2079	1081	1.09 (0.96, 1.25)	1.11 (0.97, 1.27)	1.11 (0.97, 1.27)
Quartile 3	2079	1092	1.21 (1.04, 1.40)	1.23 (1.06, 1.43)	1.23 (1.06, 1.43)
Quartile 4	2078	1108	1.38 (1.16, 1.65)	1.35 (1.12, 1.61)	1.35 (1.12, 1.62)
*P* for trend‖	–	–	<.001	.001	.001

BMI = body mass index, BRI = body roundness index, CI = confidence interval, OR = odds ratio, PS = perceived stress.

* Model 1: adjusted for age, sex, and BMI.

† Model 2: further adjusted for Model 1 + smoking, drinking, education level, residential region, and geographic region.

‡ Model 3: further adjusted for Model 2 + hypertension and diabetes.

§ Adjusted data are expressed as OR (95% CI).

‖ *P* for trend was obtained using multivariate logistic regression analyses.

To assess potential population heterogeneity, subgroup analyses of the association between BRI and PS were performed (Table 3). The positive correlation proved robust across the majority of demographic and lifestyle subgroups. Despite numerically larger effect sizes in specific cohorts, including men, younger individuals (<60 years), non-obese subjects (BMI < 30 kg/m^2^), nondrinkers, individuals with primary- or college-level education, county and rural residents, and those residing in eastern or northeastern regions, formal interaction tests indicated no significant effect modification (all *P* for interaction > .05). Conversely, smoking status emerged as the sole significant effect modifier (*P* for interaction = .033), driving a more pronounced association among smokers. Sensitivity analyses using a generalized linear model yielded consistent results (Table 4). When PS was treated as a continuous variable, higher BRI levels remained positively associated with PS scores. The graded relationship persisted across all models, supporting the robustness of the main findings.

**Table 3:** Stratified analyses for the association of BRI with PS risk across various subgroups.

Subgroups	Quartiles of BRI	Model 1*	Model 2†	Model 3‡	*P* for interaction
Sex					.070
Men (n = 4073)	Quartile 1 (n = 1020)	Reference§	Reference§	Reference§	
	Quartile 2 (n = 1017)	0.95 (0.79, 1.15)	1.02 (0.82, 1.26)	1.02 (0.82, 1.26)	
	Quartile 3 (n = 1018)	1.17 (0.95, 1.45)	1.31 (1.00, 1.72)	1.31 (1.00, 1.72)	
	Quartile 4 (n = 1018)	1.25 (0.97, 1.62)	1.47 (1.02, 2.12)	1.46 (1.01, 2.12)	
	*P* for trend	.032	.013	.013	
Women (n = 4242)	Quartile 1 (n = 1062)	Reference§	Reference§	Reference§	
	Quartile 2 (n = 1060)	1.13 (0.94, 1.36)	1.16 (0.93, 1.44)	1.15 (0.93, 1.43)	
	Quartile 3 (n = 1065)	1.23 (1.00, 1.51)	1.30 (0.98, 1.71)	1.28 (0.97, 1.68)	
	Quartile 4 (n = 1055)	1.31 (1.02, 1.67)	1.40 (0.96, 2.06)	1.40 (0.95, 2.05)	
	*P* for trend	.028	.072	.081	
Age, yr					.333
Age < 60 (n = 5823)	Quartile 1 (n = 1456)	Reference§	Reference§	Reference§	
	Quartile 2 (n = 1456)	1.05 (0.90, 1.23)	1.21 (1.01, 1.45)	1.21 (1.01, 1.45)	
	Quartile 3 (n = 1460)	1.18 (0.99, 1.41)	1.47 (1.16, 1.87)	1.47 (1.15, 1.86)	
	Quartile 4 (n = 1451)	1.26 (1.01, 1.57)	1.77 (1.27, 2.47)	1.77 (1.27, 2.47)	
	*P* for trend	.023	<.001	<.001	
Age ≥ 60 (n = 2492)	Quartile 1 (n = 623)	Reference§	Reference§	Reference§	
	Quartile 2 (n = 623)	1.12 (0.84, 1.49)	1.18 (0.86, 1.63)	1.17 (0.85, 1.62)	
	Quartile 3 (n = 623)	1.22 (0.91, 1.64)	1.37 (0.94, 2.00)	1.33 (0.91, 1.95)	
	Quartile 4 (n = 623)	1.52 (1.09, 2.11)	1.68 (1.02, 2.78)	1.63 (0.99, 2.70)	
	*P* for trend	.008	.037	.051	
BMI, kg/m^2^					.272
BMI < 30 (n = 7819)	Quartile 1 (n = 1959)	Reference§	Reference§	Reference§	
	Quartile 2 (n = 1952)	1.01 (0.89, 1.15)	1.21 (1.03, 1.43)	1.21 (1.03, 1.43)	
	Quartile 3 (n = 1957)	1.07 (0.94, 1.22)	1.46 (1.18, 1.80)	1.45 (1.17, 1.79)	
	Quartile 4 (n = 1951)	1.13 (0.98, 1.30)	1.78 (1.33, 2.37)	1.78 (1.33, 2.37)	
	*P* for trend	.052	<.001	<.001	
BMI ≥ 30 (n = 496)	Quartile 1 (n = 125)	Reference§	Reference§	Reference§	
	Quartile 2 (n = 123)	2.48 (0.12, 50.19)	2.13 (0.09, 49.30)	2.29 (0.10, 53.71)	
	Quartile 3 (n = 124)	0.41 (0.05, 3.32)	0.42 (0.04, 4.45)	0.39 (0.04, 4.14)	
	Quartile 4 (n = 124)	0.83 (0.11, 6.04)	0.93 (0.08, 10.36)	0.86 (0.08, 9.65)	
	*P* for trend	.477	.456	.517	
Smoking status					.033
Nonsmokers (n = 6089)	Quartile 1 (n = 1524)	Reference§	Reference§	Reference§	
	Quartile 2 (n = 1521)	1.03 (0.88, 1.21)	1.02 (0.87, 1.20)	1.02 (0.87, 1.20)	
	Quartile 3 (n = 1523)	1.20 (1.00, 1.43)	1.19 (0.99, 1.42)	1.18 (0.98, 1.41)	
	Quartile 4 (n = 1521)	1.36 (1.10, 1.68)	1.32 (1.07, 1.64)	1.32 (1.06, 1.64)	
	*P* for trend	.001	.004	.004	
Smokers (n = 2226)	Quartile 1 (n = 557)	Reference§	Reference§	Reference§	
	Quartile 2 (n = 556)	1.35 (1.03, 1.76)	1.39 (1.07, 1.82)	1.37 (1.05, 1.79)	
	Quartile 3 (n = 557)	1.44 (1.06, 1.96)	1.50 (1.11, 2.04)	1.49 (1.09, 2.02)	
	Quartile 4 (n = 556)	1.62 (1.10, 2.37)	1.71 (1.17, 2.52)	1.71 (1.16, 2.52)	
	*P* for trend	.020	.010	.010	
Drinking status					.345
Nondrinkers (n = 5914)	Quartile 1 (n = 1482)	Reference§	Reference§	Reference§	
	Quartile 2 (n = 1476)	1.12 (0.95, 1.32)	1.12 (0.96, 1.32)	1.12 (0.95, 1.32)	
	Quartile 3 (n = 1478)	1.27 (1.06, 1.52)	1.26 (1.05, 1.51)	1.25 (1.04, 1.50)	
	Quartile 4 (n = 1478)	1.48 (1.19, 1.83)	1.46 (1.17, 1.81)	1.46 (1.18, 1.82)	
	*P* for trend	<.001	<.001	<.001	
Drinkers (n = 2401)	Quartile 1 (n = 601)	Reference§	Reference§	Reference§	
	Quartile 2 (n = 600)	1.07 (0.82, 1.38)	1.11 (0.85, 1.44)	1.10 (0.85, 1.44)	
	Quartile 3 (n = 604)	1.21 (0.90, 1.62)	1.26 (0.94, 1.70)	1.25 (0.93, 1.68)	
	Quartile 4 (n = 596)	1.27 (0.88, 1.83)	1.32 (0.91, 1.91)	1.29 (0.89, 1.87)	
	*P* for trend	.152	.109	.134	
Geographical region					.181
Eastern (n = 2956)	Quartile 1 (n = 739)	Reference§	Reference§	Reference§	
	Quartile 2 (n = 739)	1.11 (0.88, 1.41)	1.14 (0.90, 1.45)	1.14 (0.90, 1.45)	
	Quartile 3 (n = 741)	1.26 (0.97, 1.64)	1.29 (0.99, 1.68)	1.28 (0.99, 1.67)	
	Quartile 4 (n = 737)	1.64 (1.20, 2.23)	1.69 (1.24, 2.31)	1.67 (1.22, 2.29)	
	*P* for trend	.001	<.001	.001	
Central (n = 1999)	Quartile 1 (n = 500)	Reference§	Reference§	Reference§	
	Quartile 2 (n = 500)	1.12 (0.84, 1.48)	1.15 (0.86, 1.53)	1.14 (0.86, 1.52)	
	Quartile 3 (n = 500)	1.18 (0.86, 1.61)	1.23 (0.89, 1.69)	1.22 (0.89, 1.68)	
	Quartile 4 (n = 499)	1.04 (0.71, 1.51)	1.10 (0.75, 1.63)	1.12 (0.76, 1.66)	
	*P* for trend	.798	.573	.528	
Western (n = 1950)	Quartile 1 (n = 488)	Reference§	Reference§	Reference§	
	Quartile 2 (n = 490)	0.98 (0.75, 1.29)	1.00 (0.76, 1.32)	1.02 (0.77, 1.34)	
	Quartile 3 (n = 485)	1.07 (0.78, 1.47)	1.11 (0.80, 1.52)	1.10 (0.80, 1.52)	
	Quartile 4 (n = 487)	1.02 (0.69, 1.51)	1.05 (0.71, 1.56)	1.06 (0.72, 1.58)	
	*P* for trend	.774	.657	.656	
Northeastern (n = 1410)	Quartile 1 (n = 353)	Reference§	Reference§	Reference§	
	Quartile 2 (n = 353)	1.34 (0.92, 1.95)	1.33 (0.92, 1.94)	1.32 (0.90, 1.92)	
	Quartile 3 (n = 352)	1.58 (1.05, 2.38)	1.55 (1.03, 2.33)	1.53 (1.02, 2.31)	
	Quartile 4 (n = 352)	1.85 (1.14, 3.00)	1.76 (1.08, 2.86)	1.74 (1.06, 2.84)	
	*P* for trend	.014	.026	.028	
Residential area					.527
Urban (n = 2223)	Quartile 1 (n = 556)	Reference§	Reference§	Reference§	
	Quartile 2 (n = 556)	0.99 (0.76, 1.30)	0.98 (0.75, 1.29)	0.98 (0.74, 1.28)	
	Quartile 3 (n = 557)	0.98 (0.73, 1.33)	0.98 (0.73, 1.33)	0.98 (0.72, 1.33)	
	Quartile 4 (n = 554)	1.33 (0.94, 1.88)	1.29 (0.91, 1.82)	1.28 (0.90, 1.82)	
	*P* for trend	.087	.122	.130	
Suburban (n = 1232)	Quartile 1 (n = 308)	Reference§	Reference§	Reference§	
	Quartile 2 (n = 308)	0.91 (0.63, 1.31)	0.88 (0.61, 1.27)	0.88 (0.61, 1.28)	
	Quartile 3 (n = 309)	1.04 (0.69, 1.57)	1.00 (0.66, 1.51)	1.02 (0.67, 1.54)	
	Quartile 4 (n = 307)	1.13 (0.69, 1.85)	1.03 (0.62, 1.69)	1.07 (0.65, 1.77)	
	*P* for trend	.467	.732	.613	
County (n = 1582)	Quartile 1 (n = 396)	Reference§	Reference§	Reference§	
	Quartile 2 (n = 395)	1.24 (0.90, 1.70)	1.22 (0.88, 1.69)	1.22 (0.88, 1.69)	
	Quartile 3 (n = 396)	1.47 (1.03, 2.09)	1.44 (1.01, 2.06)	1.45 (1.01, 2.07)	
	Quartile 4 (n = 395)	1.65 (1.06, 2.56)	1.58 (1.01, 2.46)	1.59 (1.02, 2.47)	
	*P* for trend	.019	.033	.031	
Rural (n = 3278)	Quartile 1 (n = 820)	Reference§	Reference§	Reference§	
	Quartile 2 (n = 823)	1.26 (1.02, 1.56)	1.25 (1.01, 1.55)	1.25 (1.01, 1.55)	
	Quartile 3 (n = 816)	1.53 (1.20, 1.96)	1.54 (1.20, 1.97)	1.53 (1.20, 1.96)	
	Quartile 4 (n = 819)	1.56 (1.15, 2.12)	1.59 (1.17, 2.17)	1.60 (1.17, 2.17)	
	*P* for trend	.002	.001	.001	
Education level					.064
Primary school (n = 1616)	Quartile 1 (n = 404)	Reference§	Reference§	Reference§	
	Quartile 2 (n = 405)	1.53 (1.08, 2.15)	1.55 (1.10, 2.19)	1.56 (1.10, 2.20)	
	Quartile 3 (n = 403)	1.68 (1.17, 2.42)	1.74 (1.21, 2.51)	1.71 (1.18, 2.47)	
	Quartile 4 (n = 404)	1.68 (1.10, 2.57)	1.77 (1.15, 2.72)	1.75 (1.14, 2.69)	
	*P* for trend	.037	.020	.025	
Junior high school (n = 3176)	Quartile 1 (n = 794)	Reference§	Reference§	Reference§	
	Quartile 2 (n = 795)	1.02 (0.82, 1.28)	1.05 (0.83, 1.31)	1.04 (0.83, 1.30)	
	Quartile 3 (n = 793)	1.12 (0.87, 1.44)	1.16 (0.90, 1.50)	1.15 (0.89, 1.48)	
	Quartile 4 (n = 794)	1.25 (0.92, 1.70)	1.31 (0.96, 1.78)	1.31 (0.96, 1.78)	
	*P* for trend	.115	.061	.064	
Senior high school (n = 1405)	Quartile 1 (n = 353)	Reference§	Reference§	Reference§	
	Quartile 2 (n = 350)	0.87 (0.62, 1.21)	0.88 (0.63, 1.23)	0.87 (0.62, 1.22)	
	Quartile 3 (n = 351)	1.08 (0.74, 1.58)	1.11 (0.76, 1.63)	1.11 (0.76, 1.63)	
	Quartile 4 (n = 351)	1.16 (0.73, 1.83)	1.21 (0.76, 1.93)	1.22 (0.76, 1.94)	
	*P* for trend	.304	.232	.216	
Vocational school (n = 790)	Quartile 1 (n = 198)	Reference§	Reference§	Reference§	
	Quartile 2 (n = 197)	0.85 (0.54, 1.34)	0.89 (0.56, 1.41)	0.91 (0.57, 1.43)	
	Quartile 3 (n = 198)	1.04 (0.61, 1.76)	1.11 (0.65, 1.89)	1.14 (0.67, 1.94)	
	Quartile 4 (n = 197)	0.87 (0.47, 1.62)	0.95 (0.50, 1.77)	0.97 (0.52, 1.82)	
	*P* for trend	.890	.908	.858	
College (n = 1275)	Quartile 1 (n = 319)	Reference§	Reference§	Reference§	
	Quartile 2 (n = 319)	1.17 (0.85, 1.62)	1.19 (0.86, 1.64)	1.18 (0.85, 1.64)	
	Quartile 3 (n = 324)	1.16 (0.80, 1.69)	1.16 (0.79, 1.70)	1.15 (0.78, 1.68)	
	Quartile 4 (n = 313)	2.02 (1.27, 3.21)	2.00 (1.26, 3.18)	1.99 (1.25, 3.17)	
	*P* for trend	.008	.010	.011	
Master’s degree or above (n = 53)	Quartile 1 (n = 14)	Reference§	Reference§	Reference§	
	Quartile 2 (n = 13)	2.27 (0.49, 10.44)	2.88 (0.60, 13.86)	2.85 (0.58, 14.13)	
	Quartile 3 (n = 13)	1.52 (0.20, 11.62)	1.62 (0.20, 12.93)	1.62 (0.20, 12.92)	
	Quartile 4 (n = 13)	Unavailable	Unavailable	Unavailable	
	*P* for trend	.669	.612	.627	

BMI = body mass index, BRI = body roundness index, CI = confidence interval, OR = odds ratio, PS = perceived stress.

* Model 1: adjusted for age, sex, and BMI.

† Model 2: further adjusted for age, sex, BMI, smoking, drinking, education level, residential region, and geographic region.

‡ Model 3: further adjusted for Model 2 + hypertension and diabetes mellitus.

§ Adjusted data are expressed as OR (95% CI).

**Table 4:** Generalized linear model analysis of the association between BRI quartiles and PS score.

N = 8315	β coefficients (95% CI)	*Z* value	*P*-value§
Model 1*			
Quartile 1	1.00 (Reference)	–	–
Quartile 2	0.022 (−0.011, 0.054)	1.32	.188
Quartile 3	0.047 (0.011, 0.084)	2.53	.012
Quartile 4	0.080 (0.036, 0.124)	3.57	<.001
Model 2†			
Quartile 1	1.00 (Reference)	–	–
Quartile 2	0.023 (−0.010, 0.055)	1.37	.171
Quartile 3	0.049 (0.013, 0.086)	2.66	.008
Quartile 4	0.078 (0.034, 0.122)	3.49	<.001
Model 3‡			
Quartile 1	1.00 (Reference)	–	–
Quartile 2	0.03 (−0.01, 0.06)	1.56	.118
Quartile 3	0.05 (0.01, 0.08)	2.59	.010
Quartile 4	0.07 (0.03, 0.11)	3.17	.002

BMI = body mass index, BRI = body roundness index, CI = confidence interval, GLMs = generalized linear models, PS = perceived stress.

* Model 1: adjusted for age, sex, and BMI.

† Model 2: further adjusted for Model 1 + smoking, drinking, education level, residential region, and geographic region.

‡ Model 3: further adjusted for Model 2 + hypertension and diabetes.

§ *P*-value was calculated using the GLMs.

**Figure 1.**
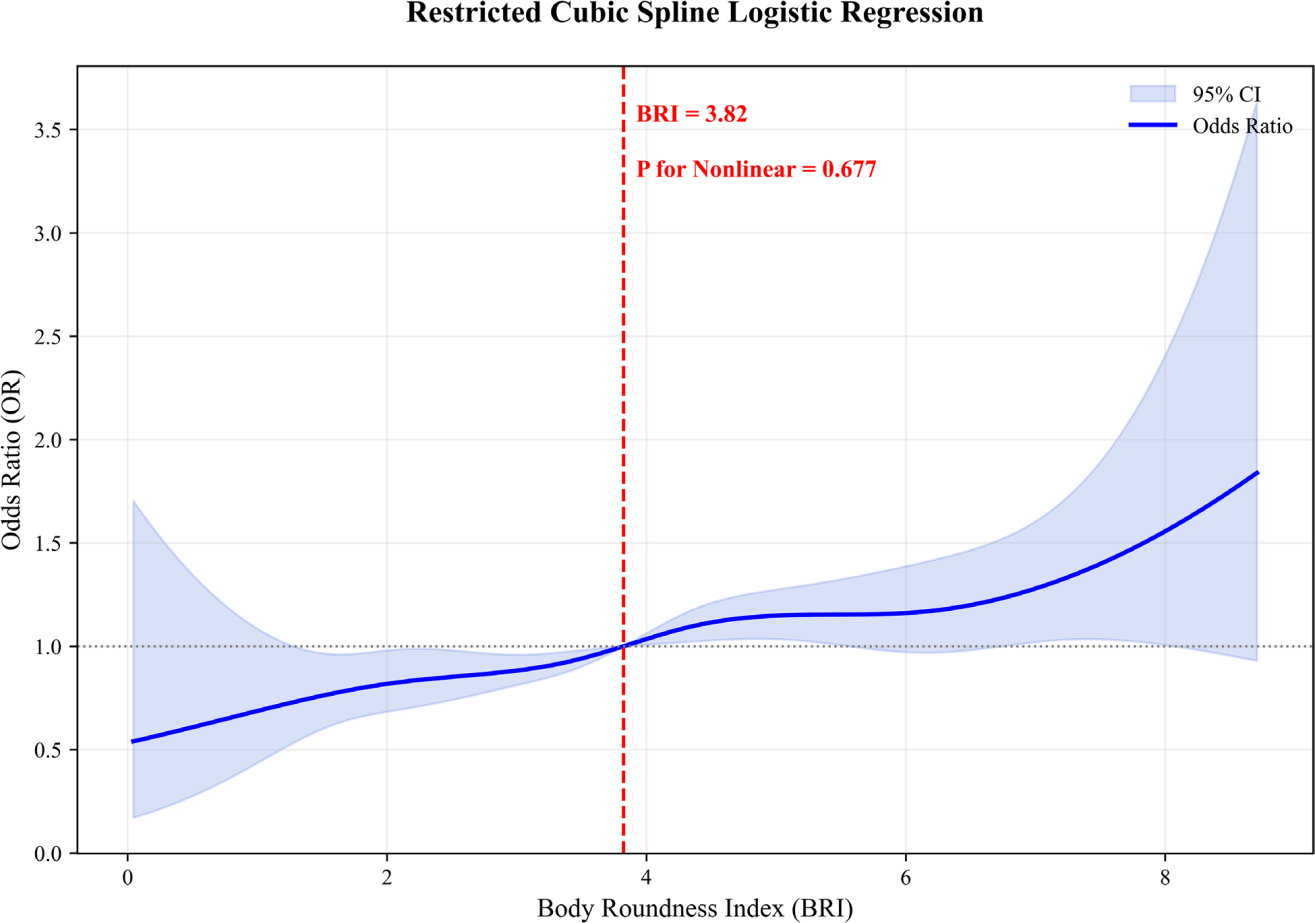
Cubic model of the association between BRI and risk of PS after adjusting for sex, age, BMI, smoking, drinking, residential area, geographical region, and education level. The graph plots the risk of PS (on the y-axis) against the measured BRI (on the x-axis). BRI = body roundness index, CI = confidence interval, PS = perceived stress.

The RCS analysis was utilized to flexibly model the dose–response relationship between BRI and PS. The analysis did not reveal significant nonlinearity (*P* for nonlinearity = .677), suggesting a linear association between BRI and PS across the observed BRI range. Specifically, increasing BRI values corresponded to progressively higher PS levels, with an apparent increase in PS risk observed at BRI ≈ 3.82 (Fig. 1). Table 5 summarizes the optimal BRI cutoff points for detecting PS, stratified by sex, age, and BMI. These cutoff values varied across subgroups, ranging from 3.832 to 5.890 in males (AUC: 0.505–0.672) and from 3.499 to 7.137 in females (AUC: 0.526–0.612). Overall, cutoff values were consistently higher in participants with BMI ≥ 30 kg/m^2^ compared with those with BMI < 30 kg/m^2^ across all sex and age categories, with a tendency for elderly females (≥60 years) in the BMI ≥ 30 kg/m^2^ group to have the highest value (7.137). In summary, the BRI demonstrated poor predictive value for PS in Chinese adults.

**Table 5:** Gender- and age-specific BRI cutoff points for screening PS levels.

Sex	Age group	BMI ≥ 30	BMI < 30
Male	≥60 yr		
	Optimal cutoff point	5.890	4.252
	AUC	0.672	0.509
	Sensitivity	0.800	0.386
	Specificity	0.544	0.632
	<60 yr		
	Optimal cutoff point	4.766	3.832
	AUC	0.573	0.505
	Sensitivity	0.939	0.432
	Specificity	0.206	0.577
Female	≥60 yr		
	Optimal cutoff point	7.137	4.092
	AUC	0.612	0.531
	Sensitivity	0.444	0.622
	Specificity	0.781	0.44
	<60 yr		
	Optimal cutoff point	6.628	3.499
	AUC	0.538	0.526
	Sensitivity	0.286	0.531
	Specificity	0.790	0.521

AUC = area under the curve, BRI = body roundness index, PS = perceived stress.

## 4. Discussion

In this cross-sectional analysis based on a nationally representative Chinese population, we observed a positive association between BRI and PS. Multivariable-adjusted models suggested that higher BRI levels were independently associated with elevated PS. Importantly, RCS analysis indicated a linear dose–response pattern (*P* for nonlinearity = .677), suggesting a gradual increase in PS levels across the range of BRI values. Subgroup analyses indicated that the association between BRI and PS was largely consistent across demographic and lifestyle characteristics. No significant interactions were observed except for smoking status (*P* for interaction = .033), with stronger associations identified among smokers. Overall, our findings suggest that BRI may be a useful anthropometric indicator associated with PS in a population-based setting.

Several biological mechanisms may explain the association between BRI and PS. These include disturbances in cortisol regulation and chronic low-grade inflammation. BRI strongly represents central adiposity, especially visceral fat accumulation, which actively influences metabolic processes and contributes to hypothalamic–pituitary–adrenal axis dysregulation.^[16]^ Excess visceral adiposity may alter glucocorticoid metabolism and impair hypothalamic–pituitary–adrenal axis negative feedback mechanisms, causing sustained or abnormal cortisol secretion.^[16]^ Chronic elevation of cortisol can affect brain regions responsible for emotional regulation, thereby increasing sensitivity to stress and perceived psychological burden.^[23]^ Moreover, central adiposity involves chronic, low-level systemic inflammation.^[24]^ Visceral adipose tissue releases pro-inflammatory cytokines, which can cross the blood–brain barrier and influence neural pathways associated with mood and stress responses.^[25]^ Persistent inflammation may disrupt neuroendocrine balance, reducing psychological resilience.^[25]^ Collectively, abnormal cortisol regulation and chronic inflammatory responses likely represent essential psychometabolic pathways linking increased BRI to elevated PS, providing biological plausibility for the observed linear dose–response relationship.^[26]^

BRI has been widely recognized as a predictor of metabolic syndrome and cardiometabolic disorders in population-based studies.^[27]^ In the present study, RCS analysis suggested that the likelihood of elevated PS increased when BRI exceeded 3.82, indicating a potential nonlinear association between BRI and PS. However, receiver operating characteristic analysis demonstrated poor discriminatory performance, with AUC values close to random classification (0.52–0.55). Thus, while BRI was significantly associated with PS at the population level, these findings should not be interpreted as evidence of effective screening capability. Moreover, the observed threshold should be considered exploratory rather than a clinically applicable cutoff. Future longitudinal studies are warranted to determine the reproducibility of these findings.

## 5. Limitations

This study is also subject to some inevitable limitations. First, one of the limitations of this study lies in its single-point cross-sectional design, which makes it challenging to ascertain the causal relationship between BRI and PS. Second, employing the median PS score as the definition of PS might result in information loss, diminished statistical power, and the concealment of potential dose–response relationships when the disparities between the 2 groups are, in fact, minor, potentially attenuating the observed association. Future research can verify clinical cutoff values to enhance the diagnostic accuracy of PS assessment, which would augment the sensitivity and specificity for identifying populations at high stress risk. Third, despite adjustment for several demographic and lifestyle covariates, residual confounding cannot be completely ruled out. Several potentially important factors, including physical activity, socioeconomic status, sleep quality, dietary habits, psychiatric comorbidities, and medication use, were unavailable or had substantial missing data and therefore were not included in the present analyses. Previous studies have shown that these factors may influence both BRI and PS. For example, lower levels of physical activity,^[28]^ poorer sleep quality,^[29]^ adverse socioeconomic conditions,^[30]^ and unhealthy dietary patterns^[31]^ have been associated with greater BRI and increased PS. Similarly, psychiatric comorbidities^[32]^ and medication use^[32]^ may affect both BRI and PS. Therefore, part of the observed association between BRI and PS may be attributable to residual confounding, and the findings should be interpreted cautiously. Fourth, measurement bias may be present. The BRI, as an anthropometric measure, provides an indirect estimate of visceral fat rather than a direct assessment through imaging techniques. In addition, the use of self-reported data for PS could introduce reporting errors, and collecting these data via questionnaires may also result in recall bias. Fifth, given the large number of subgroup analyses conducted, the possibility of type I error due to multiple comparisons cannot be excluded. Therefore, these analyses should be interpreted as exploratory and hypothesis-generating rather than confirmatory.

## 6. Conclusion

This study demonstrates a positive association between elevated BRI and PS among Chinese adults, suggesting a potential relationship between visceral adiposity and psychological stress. However, its discriminative ability was limited, suggesting that BRI alone is unlikely to effectively distinguish individuals with and without PS. Thus, the observed association should not be interpreted as evidence of effective screening capability. Further studies are needed to validate these findings in diverse populations. Prospective longitudinal research is also warranted to clarify the temporal nature of this association and to explore the biological mechanisms potentially linking adiposity, inflammation, and PS.

## Author contributions

**Conceptualization:** Naihe Zhang.

**Data curation:** Naihe Zhang.

**Formal analysis:** Naihe Zhang.

**Visualization:** Naihe Zhang.

**Writing – original draft:** Naihe Zhang.

**Writing – review & editing:** Naihe Zhang.
